# Sex differences and clinical outcomes, including ventricular tachyarrhythmias, of patients with heart failure with reduced ejection fraction treated with sacubitril/valsartan

**DOI:** 10.3389/fcvm.2024.1503414

**Published:** 2024-12-19

**Authors:** Mohammad Abumayyaleh, Carina Krack, Jonathan Demmer, Christina Pilsinger, Tobias Schupp, Michael Behnes, Katherine Sattler, Ibrahim El-Battrawy, Nazha Hamdani, Ibrahim Akin

**Affiliations:** ^1^ Department of Cardiology, Angiology, Haemostaseology and Medical Intensive Care, Medical Faculty Mannheim, University Medical Center Mannheim, Heidelberg University, Mannheim, Germany; ^2^ Department of Molecular and Experimental Cardiology, Institut für Forschung und Lehre (IFL), Ruhr-University Bochum, Bochum, Germany; ^3^ Department of Cellular and Translational Physiology and Institut für Forschung und Lehre (IFL), Molecular and Experimental Cardiology, Institute of Physiology, Ruhr-University Bochum, Bochum, Germany

**Keywords:** sex, women, men, sacubitril/valsartan, ARNI, ventricular tachyarrhythmias

## Abstract

**Background:**

Women with heart failure with reduced ejection fraction (HFrEF) often experience worse clinical outcomes compared to men, including higher rates of mortality, hospitalization, and congestion. However, the effects of sacubitril/valsartan on these outcomes, as well as on ventricular tachyarrhythmias, have not been well studied in women with HFrEF.

**Methods:**

This study included consecutive series of patients treated with sacubitril/valsartan at University Hospital Mannheim from 2016 to 2020. Baseline and follow-up data were compared between women and men. The endpoints included all-cause mortality, ventricular tachyarrhythmias, all-cause hospitalization, and congestion.

**Results:**

A total of 246 patients were analyzed, comprising 50 (20.3%) women and 196 (79.7%) men. The study population consisted of 34.3% ambulatory patients and 65.7% hospitalized patients admitted for acute decompensated or symptomatic HF. The sex distribution was as follows: among women, 48.6% were ambulatory and 51.4% were hospitalized, while among men, 30.6% were ambulatory and 69.4% were hospitalized. Ischemic cardiomyopathy (ICM) was less common as a cause of heart failure (HF) in women than in men (32% vs. 57.7%, *p* = 0.001). During the 12-month follow-up, left ventricular ejection fraction (LVEF) improved more significantly in women than in men, increasing from 29.0% (10.0–45.0) to 40.0% (15.0–59.0) in women (*p* = 0.009) compared to an increase from 28.0% (3.0–65.0) to 33.0% (13.0–60.0) in men. There were no significant differences in all-cause mortality at 12-month between women and men (4% vs. 6.7%; *p* = 0.742). The results indicated no significant differences between the sexes in the incidence of ventricular tachyarrhythmias [ventricular fibrillation [VF] and sustained ventricular tachycardia [VT]] (4.5% vs. 0.6%; *p* = 0.121) (2.3% vs. 3.9%; *p* = 1.00), hospitalizations (70.2% vs. 67.8%; *p* = 0.769), congestion at 12-month follow-up (11.4% vs. 10.1%; *p* = 0.762). Female sex was not identified as a predictor for the occurrence of ventricular tachyarrhythmias or mortality rate at 12 months [hazard ratio (HR), 0.586; 95%-confidence interval (CI) 0.17–2.016; *p* = 0.397] (HR, 1.898; 95%-CI 0.381–9.464; *p* = 0.434).

**Conclusion:**

Women with HFrEF treated with sacubitril/valsartan showed a greater improvement in LVEF compared to men, though clinical outcomes were similar across sexes. Female sex was not a predictor of ventricular tachyarrhythmias or mortality at 12 months.

## Introduction

The treatment of heart failure with reduced ejection fraction (HFrEF) has changed within the last years, including the advent of the combination drug sacubitril/valsartan (1–5). In the PARADIGM-HF trial, the combination of the neprilysin inhibitor sacubitril with the angiotensin receptor antagonist valsartan demonstrated a significant reduction in the occurrence of the composite endpoint, which consisted of death, death from cardiovascular cause, and the need for hospitalization for heart failure (HF), when compared to the treatment with enalapril alone (6). While women have a lower risk of developing HFrEF, men have an 84% higher relative risk (7). In addition, women have a lower risk of first HF hospitalization, cardiovascular death, and fewer comorbidities compared to men. Overall, there is an underuse of heart failure-specific or cardiovascular drugs such as angiotensin-converting enzyme inhibitors (ACEIs), statins, aspirin, and anticoagulants, as well as implantable devices in women with chronic heart failure (8). It remains unclear whether treatment with sacubitril/valsartan might improve the outcome of women and men with HFrEF to the same extent in a real-world setting. One study based on a real-world setting demonstrated a higher number of treatment discontinuations in women yet did not address the clinical outcome (9). A recent pooled analysis of the data from the PARADIMG-HF and the PARAGON-HF studies indicated that women may benefit from treatment, not only at low, but also at high levels of the LVEF (10). However, real-world data on treatment response to sacubitril/valsartan by sex are limited. This analysis shows real-world data in the treatment of HFrEF patients with sacubitril/valsartan according to sex. We also compared clinical outcomes including death from any cause, ventricular tachyarrhythmias, all-cause hospitalization, and congestion at 12 months.

## Methods

A total of 245 consecutive patients were included in the study at the Department of Cardiology, Angiology, Haemostaseology and Medical Intensive Care, University Medical Center Mannheim, Heidelberg University. These patients were diagnosed with HFrEF in accordance with the 2016 European Society of Cardiology Guidelines (ESC) (11). The inclusion criteria were as follows: (1) The presence of heart failure symptoms, as defined by the New York Heart Association (NYHA) functional classification system, in patients who have been optimally treated with ACEIs or angiotensin-II-receptor blockers (ARBs), beta-blockers, and mineralocorticoid receptor antagonists (MCRA); (2) left ventricular ejection fraction (LVEF) ≤40%; (3) implantation of cardioverter-defibrillator (ICD) or cardiac resynchronization therapy (CRT); and (4) receipt and tolerance of sacubitril/valsartan. Initially, patients received a dose of 24/26 mg twice daily, which was increased to 97/103 mg twice daily over a period of 3–6 weeks, contingent on tolerability.

This study was conducted in accordance with the Declaration of Helsinki regarding research involving human subjects and was approved by the Ethics Committee of the Medical Faculty Mannheim Heidelberg University.

### Data collection

The data and clinical outcomes of patients were gathered from the source data, assessed through chart review, and contacts with the outpatient practice at follow-up. Baseline characteristics of patients presenting before sacubitril/valsartan, including demographics, medical history, NYHA classification, clinical parameters, electrocardiogram (ECG), arrhythmias assessed by querying the ICD or CRT, cardiac devices, and medications, were available. LVEF was measured using the Simpson biplane method. Laboratory parameters were also evaluated, including potassium in mmol/L, glomerular filtration rate (GFR) in ml/min, troponin I (TNI) in ng/ml, N-terminal pro-B-type natriuretic peptide (NT-proBNP) in pg/ml, hemoglobin (Hb) in g/dl, glycosylated hemoglobin (HbA1c) in%. Arrhythmias [ventricular fibrillation (VF), non-sustained ventricular tachycardia (NSVT), and ventricular tachycardia (VT)] were also collected. Hospitalizations, congestions, and mortality were also gathered at baseline and after the initiation of sacubitril/valsartan in women as compared to men.

### Definitions

For the purposes of this study, hospitalization is defined as any readmission to our hospital. Congestion is defined as the presence of one or more fluid overload symptoms, including pulmonary rales, third heart sound, jugular venous stasis, hepatomegaly, peripheral edema, high levels of NT-proBNP, and acute depression of heart function. These symptoms are diagnosed by echocardiography and radiographic signs of decompensation.

### Primary and secondary endpoints

The primary endpoints were 12-month mortality and the occurrence of ventricular tachyarrhythmias (VF, NSVT, VT). Secondary endpoints included atrial fibrillation (AF), hospitalization, congestion, and improvement of LVEF and NYHA classification. These endpoints reflect both short- and long-term patient health and align with previous studies assessing the clinical efficacy of HF therapies.

### Statistics

The data were presented as mean ± standard deviation (SD) for continuous variables with a normal distribution, median (min–max) for continuous variables with a non-normal distribution, and frequency (%) for categorical variables. The Kolmogorov-Smirnov test was used to assess normal distribution. Continuous variables with normal and non-normal distributions were compared by student's *t*-test and Mann-Whitney-*U*-test, respectively. The chi-squared test or Fisher's exact test was employed to assess categorical variables. The Wilcoxon signed-rank test was utilized for paired nonparametric quantitative variables. The McNemar test was applied to paired qualitative variables. A *p*-value < 0.05 was deemed statistically significant. Univariate analysis was employed to identify predictors of mortality and the occurrence of ventricular tachyarrhythmias. The predictors with a *p*-value <0.05 were subjected to a multivariate regression analysis using the Cox method. The statistical analysis was conducted using the SPSS software version 27.0.

## Results

### Comparison of women and men

The patients were divided into two categories according to sex: women (*n* = 50, 20.3%) and men (*n* = 196, 79.7%). The median age of women was 71.5 years [median (min–max): 38–92 years], while that of men was 70 years (33–91 years). The mean body mass index (BMI) in kg/m^2^ (±SD) was similar in both groups, with a mean of 28.35 ± 7.12 in women and 29.33 ± 6.1 in men. The incidence of ischemic cardiomyopathy (ICM) was less in women than in men (32% vs. 57.7%, *p* = 0.001), while the incidence of non-ischemic cardiomyopathy (NICM) was higher in women than in men (70% vs. 45.9%, *p* = 0.002). Baseline characteristics, including medical history, NYHA classification, clinical parameters, arrhythmias, cardiac devices, and medication, are presented in Table 1.

**Table 1:** Baseline characteristics of hFrEF patients presenting before sacubitril/valsartan by sex.

Variables	Women *n* = 50	Men *n* = 196	*P*-value*
Demographics			
Age in years; median (min–max)	71.5 (38–92)	70 (33–91)	0.862
BMI in kg/m^2^; mean ± SD	28.35 ± 7.12	29.33 ± 6.1	0.866
Inpatient; *n* (%)	18/35 (51.4)	93/134 (69.4)	–
Outpatient; *n* (%)	17/35 (48.6)	41/134 (30.6)	–
Medical history; *n* (%)			
Smoking	21/50 (58)	130/196 (66.3)	**0** **.** **001**
Lung disease			
Asthma	3/50 (6)	3/196 (1.5)	0.1
COPD	3/50 (6)	45/196 (23)	**0** **.** **007**
Arterial hypertension	37/50 (74)	145/195 (74.4)	0.959
Diabetes mellitus	16/50 (32)	75/196 (38.3)	0.413
Positive family history	15/50 (30)	49/196 (25)	0.472
History of malignancy	12/50 (24)	30/196 (15.3)	0.145
Myocardial infarction			
STEMI	10/50 (20)	67/196 (34.2)	0.051
NSTEMI	7/50 (14)	36/196 (18.4)	0.459
Coronary heart disease	25/50 (50)	137/196 (69.9)	**0** **.** **011**
Stroke	8/50 (16)	30/196 (15.3)	0.915
Coronary bypass	5/50 (10)	37/196 (18.9)	0.133
NICM	35/50 (70)	90/196 (45.9)	**0** **.** **002**
ICM	16/50 (32)	113/196 (57.7)	**0** **.** **001**
NYHA-Classification; *n* (%)			
III	26/50 (52)	99/196 (50.5)	0.433
IV	3/50 (6)	19/196 (9.7)	0.774
Clinical parameter; median (min–max)			
Systolic BP mmHg	120 (90–180)	122 (80–190)	0.628
Diastolic BP mmHg	80 (60–120)	80 (42–112)	0.39
HR bpm	75.5 (52–123)	73.5 (46–156)	0.152
ECG; median (min–max)			
PQ in ms	157 (96–260)	176 (114–396)	**0** **.** **021**
QTc in ms	477 (407–576)	468 (207–696)	0.385
MitraClip; *n* (%)	2/50 (4)	12/196 (6.2)	0.741
Arrhythmias; *n* (%)			
AF	21/50 (42)	93/196 (47.4)	0.453
VF	4/50 (8)	20/196 (10.2)	1.000
NSVT	3/50 (6)	26/196 (13.3)	0.149
VT	3/50 (6)	25/196 (12.8)	0.2
Cardiac Device; *n* (%)			
CRT	10/50 (20)	42/196 (21.5)	0.8
S-/TV-ICD	20/50 (40)	113/196 (57.9)	0.021
PM	1/50 (2)	15/196 (7.7)	0.205
CCM	5/50 (10)	33/196 (16.9)	0.223
Vagusstimulator	0/50 (0)	1/196 (0.5)	1.00
Medication; *n* (%)			
Beta-blocker	44/50 (88)	180/196 (91.8)	0.239
ARB	11/50 (22)	49/196 (25)	0.608
Aldosterone antagonist	32/50 (64)	126/196 (64.3)	0.884
ACEI	28/50 (56)	108/196 (55.1)	0.975
Ivabradine	2/50 (4)	6/196 (3.1)	0.671
Diuretics	37/50 (74)	157/196 (80.1)	0.220
Platelet aggregation inhibitors	20/50 (40)	98/196 (50)	0.185
Anticoagulation	22/50 (44)	95/196 (48.5)	0.490
Amiodarone	4,750 (8)	24/196 (12.2)	0.376
Sotalol	0/50 (0)	1/196 (0.5)	1.00
Statin	24/50 (48)	137/196 (69.9)	**0** **.** **002**
Metformin	2/50 (4)	25/196 (12.8)	0.060
Insulin	5/50 (10)	24/196 (12.2)	0.554
SGLT2-inhibitor	1/50 (2)	11/196 (5.6)	0.126
DPP-4-inhibitor	4/50 (8)	13/196 (6.6)	0.765

*n*, number of data; SD, standard deviation; BMI, body-mass-index; COPD, chronic obstructive pulmonary disease; STEMI, ST-segment elevation myocardial infarction; NSTEMI, non-ST-segment elevation myocardial infarction; NICM, non-ischemic cardiomyopathy; ICM, ischemic cardiomyopathy; NYHA, New York Heart Association; BP, blood pressure; HR, heart rate; ECG, electrocardiogram; PQ, PQ interval; QTc, corrected QT interval; LVEF, left ventricular ejection fraction; AF, atrial fibrillation; VF, ventricular fibrillation; NSVT, non-sustained ventricular tachycardia; VT, ventricular tachycardia; CRT, cardiac resynchronization therapy; CRT-D, cardiac resynchronization therapy with defibrillator; S-ICD, subcutaneous implantable cardioverter-defibrillator; TV-ICD, transvenous implantable cardioverter-defibrillator; PM, pacemaker; CCM, cardiac contractility modulation; ARB, angiotensin II receptor blocker; ACEI, angiotensin-converting enzyme inhibitor; SGLT2, sodium-glucose transport protein 2; DPP-4, dipeptidyl peptidase 4; bold indicates significant values.

* *P*-values for the comparison between male and female patients.

### Echocardiographic values, NYHA classification, and laboratory values

**Figure 1:**
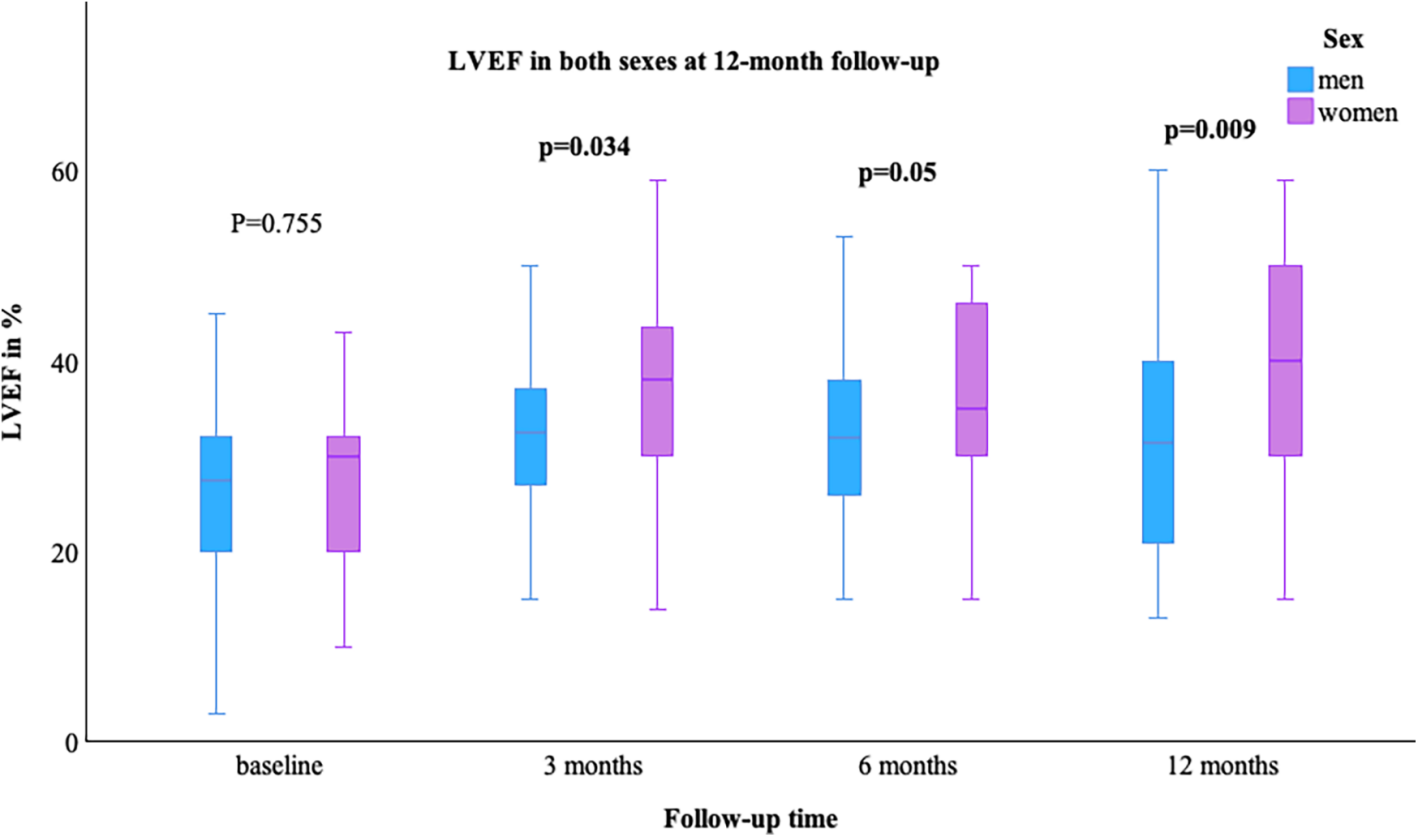
LVEF in both sexes at 12-month follow-up. LVEF, left ventricular ejection fraction; bold indicates significant values.

**Figure 2:**
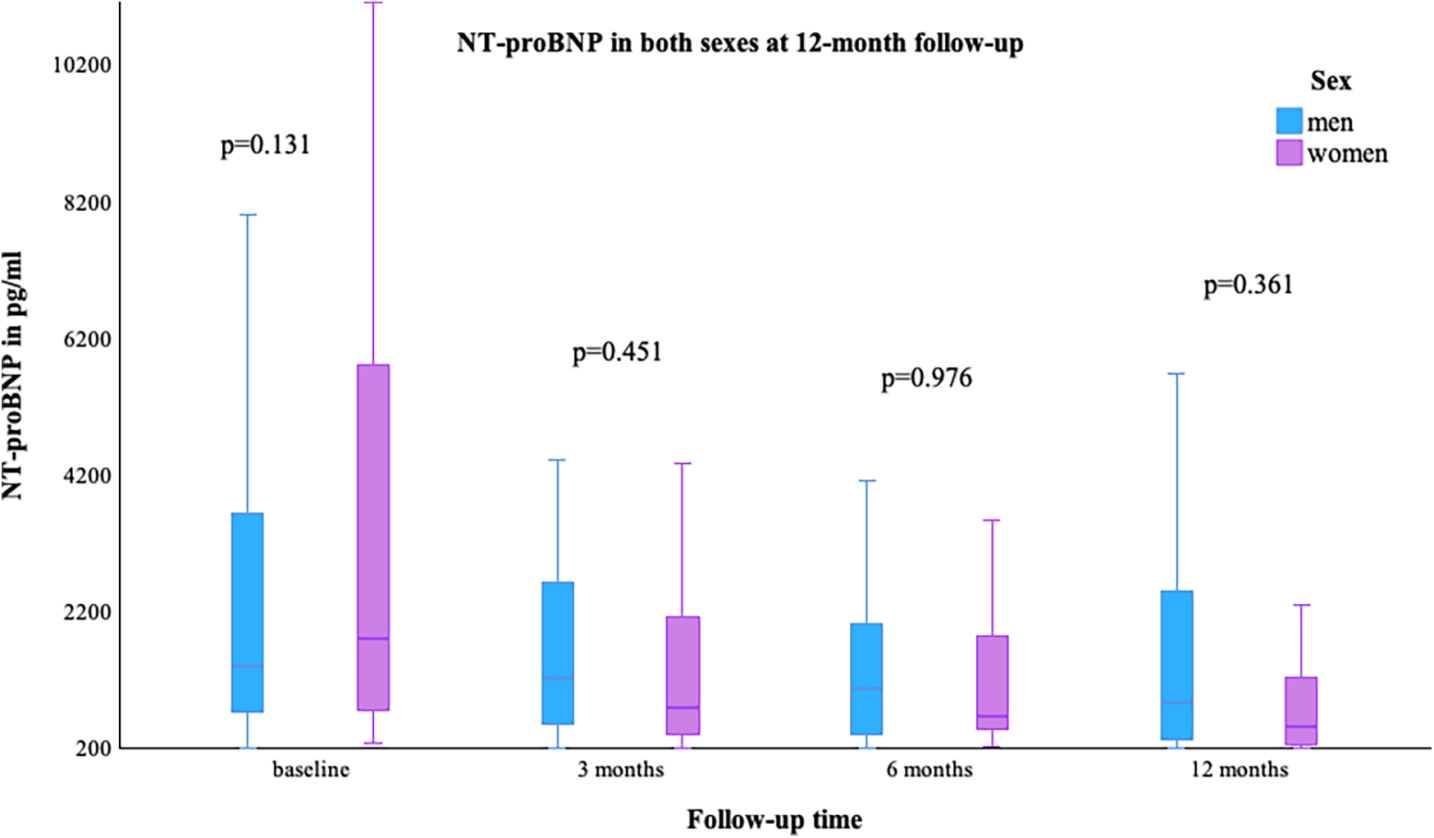
NT-proBNP in both sexes at 12-month follow-up.

The LVEF [median (min–max)] was comparable at baseline in both sexes, with a value of 29.0% (10.0–45.0) in women and 28.0% (3.0–65.0) in men; *p* = 0.755. At the 12-month follow-up, an improvement in LVEF was observed in more women than men [women 40.0% (15.0–59.0) vs. men 33.0% (13.0–60.0)], Figure 1. NYHA class improved in both groups, as shown in Table 2. The value of NT-proBNP decreased in both groups. In women, the value decreased from 2,085 pg/ml (257–26,778) to 551 pg/ml (69–6,054), while in men, it decreased from 1,339.5 pg/ml (48–74,676) to 973 pg/ml (35–8,598); *p* = 0.361, Figure 2. Table 2 presents the remaining laboratory values.

**Table 2:** Echocardiographic, LVEF improvement, laboratory, and NYHA classifications in women versus men with hFrEF at baseline and 12-month follow-up after sacubitril/valsartan.

Variables	Women	Men	*P*-value*
	50	196	
Echocardiographic values			
LVEF%; median (min–max)			
Baseline	29.0 (10.0–45.0)	28.0 (3.0–65.0)	0.755
3 months	38.0 (14.0–59.0)	33.0 (5.0–69.0)	**0** **.** **034**
6 months	37.0 (15.0–54.0)	32.0 (15.0–60.0)	**0** **.** **05**
12 months	40.0 (15.0–59.0)	33.0 (13.0–60.0)	**0** **.** **009**
LVEF improvement^a^; *n* (%)			
NICM	24/26 (92.3)	42/90 (46.7)	**0** **.** **009**
ICM	2/26 (7.7)	48/90 (53.3)	0.395
NYHA classification			
III; *n* (%)			
Baseline	26/40 (65)	98/169 (58)	0.433
3 months	13/36 (36.1)	45/143 (31.5)	0.634
6 months	14/35 (40)	39/139 (28.1)	0.19
12 months	9/36 (25)	38/134 (28.4)	0.645
IV; *n* (%)			
Baseline	3/40 (7,5)	19/169 (11.2)	0.774
3 months	2/36 (5.6)	8/143 (5.6)	1.00
6 months; *n* (%)	0/35 (0)	6/139 (4.3)	0.601
12 months; *n* (%)	0/36 (0)	3/134 (2.2)	1.00
Laboratory values			
Potassium mmol/L; median (min–max)			
Baseline	4.04 (3–6.5)	4.2 (2.1–5.7)	0.081
3 months	4.19 (3–6)	4.3 (2.9–5.36)	0.234
6 months	4.3 (3.2–5.43)	4.3 (2.7–5.6)	0.952
12 months	4.33 (3.51–5.7)	4.2 (2.86–5.7)	0.156
GFR ml/min; median (min–max)			
Baseline	56 (26–110)	54.4 (10–128)	0.919
3 months	51 (27–95.6)	51.5 (21–117)	0.949
6 months	43 (19–100)	53.5 (3–128.8)	0.244
12 months	49 (11–95)	53 (14–102)	0.588
TNI ng/ml; median (min–max)			
Baseline	0.093 (0.009–71.85)	0.06 (0.013–138.69)	0.625
3 months	0.015 (0.002–0.78)	0.021 (0.014–0.302)	0.205
6 months	0.039 (0.015–0.07)	0.05 (0.013–0.88)	0.474
12 months	0.019 (0.015–0.054)	0.015 (0.005–14.94)	0.658
NT-proBNP pg/ml; median (min–max)			
Baseline	2,085 (257–26,778)	1,339.5 (48–74,676)	0.131
3 months	668 (139–15,505)	1,159 (31–13,324)	0.451
6 months	657 (193–26,041)	951.5 (78–5,210)	0.976
12 months	551 (69–6,054)	973 (35–8,598)	0.361
Hb g/dl; median (min–max)			
Baseline	13.45 (8.2–17)	13.9 (8.2–17.8)	**0** **.** **034**
3 months	13.3 (9.4–16.4)	13.7 (8.8–18.3)	0.058
6 months	13 (11.1–15.7)	13.8 (9.3–17.6)	**0** **.** **03**
12 months	13.1 (9–17.1)	13.9 (7.1–17.2)	0.058
HbA1c%; median (min–max)			
Baseline	6.1 (4.3–9.7)	6.2 (4.7–11.5)	0.61
3 months	5.95 (5.2–8.7)	6.8 (5.2–12.2)	0.121
6 months	7.4 (5.5–45.1)	6.5 (5.3–11.7)	0.303
12 months	6 (5.3–14.4)	6.2 (5.2–11.4)	0.435

*n*, number of data; LVEF, left ventricular ejection fraction; NICM, non-ischemic cardiomyopathy; ICM, ischemic cardiomyopathy; NYHA, New York Heart Association; GFR, glomerular filtration rate; NT-proBNP, N-terminal pro-B-type natriuretic peptide; Hb, hemoglobin; HbA1c, glycosylated hemoglobin; bold indicates significant values.

^a^ An improvement in LVEF was defined as an increase of ≥5% in LVEF at 12-month follow-up.

* *P*-values for the comparison between subgroups.

### Clinical outcomes

The mortality rate (primary endpoint) was comparable in both sexes at the 12-month follow-up (4% vs. 6.7%; *p* = 0.742). Ventricular tachyarrhythmias at the 12-month follow-up were comparable between the two groups (VF: 4.5% vs. 0.6%; *p* = 0.121; VT: 2.3% vs. 3.9%; *p* = 1.00). Hospitalization and congestion at the 12-month follow-up were also comparable in both sexes following the initiation of sacubitril/valsartan, as shown in Table 3.

**Table 3:** Clinical outcomes in women as compared to men with hFrEF at 12-month follow-up.

Variables	Women	Men	*P*-value*
Mortality; *n* (%)			
Baseline	0/50 (0)	2/195 (1)	1.00
3 months	2/50 (4)	6/195 (3.1)	1.00
6 months	0/50 (0)	5/195 (2.6)	0.742
Total mortality rate at 12 months	2/50 (4)	13/195 (6.7)	0.742
Hospitalization; *n* (%)			
Baseline	26/50 (52)	117/193 (60.6)	0.335
3 months	15/49 (30.6)	81/192 (42.2)	0.147
6 months	32/47 (69.1)	105/185 (56.8)	0.167
12 months	33/47 (70.2)	120/177 (67.8)	0.769
Congestion; *n* (%)			
Baseline	14/43 (32.6)	65/183 (35.5)	0.732
3 months	3/39 (7.7)	16/151 (10.6)	0.769
6 months	2/34 (5.9)	12/147 (8.2)	1.00
12 months	4/35 (11.4)	14/138 (10.1)	0.762
Atrial fibrillation; *n* (%)			
Baseline	21/50 (42)	93/194 (47.9)	0.472
3 months	1/50 (2)	3/182 (1.6)	1.00
6 months	1/43 (2.3)	1/170 (0.6)	0.362
12 months	2/44 (4.5)	6/162 (3.7)	0.679
Ventricular fibrillation; *n* (%)			
Baseline	4/50 (8)	20/194 (10.3)	0.793
3 months	1/50 (2)	4/177 (2.3)	0.58
6 months	1/39 (2.6)	3/149 (2.)	1.00
12 months	2/44 (4.5)	1/156 (0.6)	0.121
Non sustained ventricular tachycardia; *n* (%)			
Baseline	3/50 (6)	26/194 (13.4)	0.152
3 months	1/49 (2)	8/181 (4.4)	0.688
6 months	1/43 (2.3)	10/171 (5.8)	0.698
12 months	0/44 (0)	4/158 (2.5)	0.579
Sustained ventricular tachycardia; *n* (%)			
Baseline	3/50 (6)	25/194 (12.9)	0.176
3 months	1/50 (2)	3/181 (1.7)	1.00
6 months	1/43 (2.3)	3/171 (1.8)	1.00
12 months	1/43 (2.3)	6/155 (3.9)	1.00

*n*, number of data.

* *P*-values for the comparison between subgroups.

### Predictor of ventricular tachyarrhythmias and mortality

Female sex was not identified as a predictor for the occurrence of ventricular tachyarrhythmias or mortality rate at 12 months; hazard ratio (HR) 0.586 [95% confidence interval (CI) 0.17–2.016; *p* = 0.397] for ventricular tachyarrhythmias; HR 1.898 (95% CI 0.381–9.464; *p* = 0.434) for mortality rate. However, amiodarone was identified as a negative predictor for the occurrence of ventricular tachyarrhythmias at the 12-month follow-up (HR 11.402 (95% CI 2.313–60.567; *p* = 0.017), Tables 4, 5.

**Table 4:** Predictors of ventricular tachyarrhythmias at 12-month follow-up.

Variable	Univariate analysis			Multivariate analysis		
	HR	95% CI	*P*-value	HR	95% CI	*P*-value
Patient characteristics						
Age >65	0.568	0.228–1.415	0.225			
Female as sex	0.586	0.17–2.016	0.397			
Medical history						
BMI ≥30	0.837	0.26–2.69	0.765			
Smoking	0.984	0.386–2.506	0.972			
COPD	1.42	0.465–4.337	0.538			
Arterial hypertension	0.53	0.186–1.506	0.233			
DM type II	0.764	0.327–1.786	0.535			
Positive family history	1.333	0.441–4.028	0.611			
History of malignancy	0.717	0.21–2.452	0.596			
Cardiac decompensation	1.259	0.403–3.933	0.692			
Coronary heart disease	0.372	0.119–1.161	0.089			
Stroke	2.067	0.655–6.517	0.215			
Coronary bypass	1.909	0.756–4.819	0.171			
Drugs on admission						
Beta-blocker	0.995	0.227–4.368	0.995			
ARB	0.974	0.393–2.414	0.954			
Aldosterone antagonist	1.019	0.388–3.066	0.974			
ACEI	0.819	0.342–1.962	0.655			
Diuretics	1.362	0.453–4.097	0.583			
Platelet aggregation inhibitors	0.759	0.312–1.844	0.542			
Anticoagulation	0.817	0.327–2.038	0.664			
Amiodarone	9.805	1.606–59.884	**0** **.** **013**	11.402	2.313–60.567	**0.017**
Statin	0.751	0.286–1.972	0.561			
Metformin	1.078	0.389–2.985	0.8,851			
Insulin	0.515	0.148–1.792	0.297			
SGLT2-inhibitor	2.486	0.305–20.242	0.395			
DPP-4-inhibitor	0.891	0.201–3.958	0.881			

HR, hazard ratio; CI, confidence interval; NICM, non-ischemic cardiomyopathy; ICM, ischemic cardiomyopathy; BMI, body-mass-index; COPD, chronic obstructive pulmonary disease; ARB, angiotensin II receptor blocker; ACEI, angiotensin-converting enzyme inhibitor; SGLT2, sodium-glucose transport protein 2; DPP-4, dipeptidyl peptidase 4; bold indicates significant results.

**Table 5:** Predictors of 12-month mortality.

Variable	Univariate analysis		
	HR	95% CI	*P*-value
Patient characteristics			
Age >65	0.154	0.014–1.703	0.127
Female as sex	1.898	0.381–9.464	0.434
NICM	0.555	0.182–1.691	0.301
ICM	1.467	0.503–4.28	0.482
Medical history			
BMI ≥30	3.533	0.973–13.32	0.062
Smoking	1.843	0.635–5.351	0.261
COPD	0.968	0.26–3.603	0.961
Arterial hypertension	0.672	0.139–3.257	0.622
DM type II	2.498	0.678–9.204	0.169
Positive family history	1.683	0.444–6.382	0.444
History of malignancy	0.776	0.212–2.836	0.701
Cardiac decompensation	2.111	0.453–9.872	0.341
Coronary heart disease	4.128	0.542–32.526	0.178
Stroke	0.571	0.175–1.857	0.351
Coronary bypass	0.909	0.281–2.944	0.873
Arrhythmia before sacubitril/valsartan			
AF	0.614	0.211–1.788	0.371
NSVT	0.811	0.252–2.614	0.726
Drugs on admission			
ARB	1.55	0.483–4.98	0.461
Aldosterone antagonist	1.393	0.475–4.082	0.546
ACEI	1.488	0.511–4.331	0.466
Ivabradine	0.501	0.063–3.981	0.513
Platelet aggregation inhibitors	1.09	0.371–3.205	0.875
Anticoagulation	0.753	0.262–2.168	0.599
Statin	0.892	0.266–2.986	0.852
Insulin	2.646	0.654–10.706	0.172
DPP-4-inhibitor	0.657	0.139–3.102	0.596

HR, hazard ratio; CI, confidence interval; NICM, non-ischemic cardiomyopathy; ICM, ischemic cardiomyopathy; BMI, body-mass-index; COPD, chronic obstructive pulmonary disease; DM, diabetes mellitus; AF, atrial fibrillation; NSVT, non-sustained ventricular tachycardia; ARB, angiotensin II receptor blocker; ACEI, angiotensin-converting enzyme inhibitor; DPP-4, dipeptidyl peptidase 4.

## Discussion

The present study examines the impact of sacubitril/valsartan on women and men over a 12-month period. The primary findings of this study are as follows: (1) Female sex was not identified as a predictor for the occurrence of ventricular tachyarrhythmias or mortality. (2) Mortality, ventricular tachyarrhythmias, hospitalization, and congestion were comparable in both sexes. (3) There was a significantly greater improvement in LVEF in women than in men.

Sex differences represent a traditional risk factor, as well as sex-specific risk factors that influence the prevalence and manifestation of HF in unique ways. Ischemic heart disease, which results in myocardial dysfunction and ICM, is the primary cause of HFrEF. The majority of patients with HFrEF are men, while women tend to display heart failure with preserved ejection fraction (HFpEF) (12). The reasons for this finding remain unclear. Several biological, epidemiological, and clinical characteristics are discussed in generating this observation. Among these are differences in heart morphology and structure of women and men, a lower incidence of ischemic heart disease but a higher mortality rate due to acute myocardial ischemia in women, an influence of sex hormones and their receptors, and finally, an influence of the maternally inherited mitochondria on the cardiovascular system (12). All of them being in part the reason for the Yentl Syndrome. The unequal distribution of sexes among patients with HFrEF is exemplified by the fact that even the large PARADIGM-HF study, which introduced sacubitril/valsartan as a treatment for HFrEF, demonstrated an unequal number of women and men (13). In our study, fewer women than men were present, although the enrollment process specifically included each patient starting on sacubitril/valsartan in our hospital. In accordance with the aforementioned primary etiology of HFrEF, our study revealed that coronary artery disease and ICM were less prevalent in women than in men. Despite the potential efficacy of primary percutaneous coronary intervention (PCI) and novel thienopyridines in the treatment of ICM, NICM may result from tachycardiomyopathy, myocarditis, or other potentially reversible conditions with higher recovery rates. This also explains the observed differences in LVEF improvement, with women demonstrating a higher rate of improvement than men. Furthermore, we postulated that the prevalence of scar tissue in ICM patients is higher than in NICM. Consequently, the improvement in LVEF observed in women is attributable to a higher incidence of NICM in this group. Previous study has also reported that patients with HFrEF showed an improvement in LVEF after the initiation of sacubitril/valsartan therapy (14). Notably, in a smaller subset of our cohort from a prior study, women demonstrated greater LVEF improvement than men at the 12-month follow-up (15). While another study has identified female sex as an independent predictor of functional class improvement, our findings do not support this association (16).

The clinical outcomes, including mortality and hospitalization, were found to be comparable in both groups, as corroborated by the findings of our study (17). In a meta-analysis, women and men with HFrEF exhibited similar rates of all-cause mortality, cardiovascular mortality, and HF hospitalizations (17). A different study found that women with HFrEF were more likely to be hospitalized and to have NYHA classes III or IV than men while being treated with ACEI and ARB, but not sacubitril/valsartan. Furthermore, other comorbidities were observed in women, including anemia (12). Registry data indicated that female sex was a predictor of functional class improvement (16). However, Dewan et al. reported that women with HFrEF exhibited a more unfavorable outcome than men (8). Nevertheless, further studies with a larger number of women are required to ascertain the long-term outcomes of women with HFrEF.

Regarding the risk of ventricular tachyarrhythmias, female sex was not identified as a predictor. In general, it has been reported that the initiation of sacubitril/valsartan was associated with a lower degree of VT/VF, resulting in a reduction in the number of ICD interventions (18). Moreover, sacubitril/valsartan was found to reduce ventricular arrhythmias and appropriate ICD shocks as well as cardiac arrest in patients with HFrEF compared to ACEIs and ARBs (19–23). Another study demonstrated that NICM patients with reduced EF and ICD undergoing sacubitril/valsartan treatment experienced a reduction in the incidence of both atrial and ventricular arrhythmias, along with an improvement in ICD atrial electrical parameters (24). In addition, sacubitril/valsartan was found to be associated with a reduction in acute systemic inflammatory markers, as well as a reduction in total scar and border zone mass on late gadolinium-enhanced magnetic resonance imaging with decreasing the risk of ventricular arrhythmias (25). However, other studies have reported no effect of sacubitril/valsartan on arrhythmias (26). The beneficial effect on ventricular arrhythmias may be attributed to a direct pharmacological effect of sacubitril/valsartan on cardiac reverse remodeling and/or small-conductance Ca2+-activated potassium channel type 2 (KCNN2)-associated electrical remodeling (27, 28). However, data on sex differences in HFrEF are limited.

Summarizing, the long-term mortality rate was comparable between women and men. The improvement in LVEF observed in women may be attributed to a variety of factors, including different etiologies. However, it is also possible that other, as yet unknown, reasons may be responsible.

## Conclusion

This study demonstrated the effectiveness of sacubitril/valsartan in patients with HFrEF, regardless of sex. Moreover, female sex was not identified as a predictor of ventricular tachyarrhythmias or mortality. However, LVEF showed a more pronounced improvement in women, which may be due to the higher prevalence of NICM in this group.

## Study limitations

This study is a retrospective single-center study with a limited follow-up period and a small number of patients. LVEF was not systematically evaluated using, for example, cardiac magnetic resonance tomography. NYHA class was evaluated without using a qualitative evaluation questionnaire. Some patients did not receive the target dose in ambulatory practice. LVEF was evaluated by the same cardiologists to reduce the intra- and inter-observer variability. Therefore, caution is required when interpreting the data. Furthermore, it is not possible to exclude the possibility of bias due to unknown confounders, given the retrospective nature of the study. The documentation of arrhythmias was conducted via device interrogation. However, this study represents real-world data, which provides valuable insights into the efficacy of sacubitril/valsartan in clinical practice.

## Data Availability

The original contributions presented in the study are included in the article/Supplementary Material, further inquiries can be directed to the corresponding author.
